# Collectanea Medica, Consisting of Anecdotes, Facts, Extracts, Illustrations, Queries, Suggestions, &c.

**Published:** 1815-11

**Authors:** 


### COLLECTANEA MEDICA,

CONSISTING OF

### ANECDOTES, FACTS, EXTRACTS, ILLUSTRATIONS, QUERIES, SUGGESTIONS, &c.

RELATING TO THE

*History or the Art of Medicine, and the Auxiliary Sciences.*

---

*Memoir on the State and Composition of the Atmosphere, considered as the Cause of the Hospital Gangrene, or Putrescence among the Wounded.* By Professor S. J. Brugmans, of Leyden.*

SECTION I. *Developement of the Question.*—In order to make known precisely the scope of the question proposed, it will be necessary to quote, from the programme of the society for 1813, the following passage:—

As we observe very frequently, particularly in military hospitals, that sudden and rapid mortification, called in Latin *gangræna nosocomialis,* and in Germany denominated the *hospitaal brand*, that the disease often attacks almost all the wounded who are in the same apartment; that several individuals die, notwithstanding the application of the curative means and treatment, successfully resorted to in the other species of gangrene; and, finally, that the causes of this kind of mortification are still involved in much obscurity; and it is, therefore, required to know—

"Are there any physical or chemical methods of ascertaining the state and composition of the atmosphere which are the immediate cause of the hospital gangrene or putrescence?—Of what do these causes consist, and how are they produced?—What are the means best calculated to prevent this state of the atmosphere, or rather to correct it as speedily and efficaciously as possible, if it already exists?"

The society is desirous that the solution of this question should lay the foundation of a more precise and more perspicuous medico-chirurgical theory of the hospital gangrene.

§ 2. Having had an opportunity, for several years, of seeing, under various circumstances, the ravages of hospital gangrene in a great number of patients in various hospitals,† having for a long time been actually engaged in collecting abundance of minute observations, and even of instituting experiments in order to elucidate the most obscure points; I take the liberty to offer to the society

---

* This paper obtained the prize given by the society of Sciences at Harlem, at their anniversary meeting, May 21, 1814.

† The names of living individuals, as well as of the places where the hospitals here alluded to are situated, were added, when this paper first appeared.

a succinct a succinct recapitulation of my labours, which are now submitted to their judgment.

Although the society requires only a treatise on the state and composition of the atmosphere when charged with the miasma of the hospital gangrene, the physical and chemical researches of the cause are so intimately connected with the medical considerations on the effect produced, that I am under the necessity of saying something on the disease itself, omitting every thing that relates to the medico-chirurgical treatment properly so called; may I be allowed to divide my memoir into four chapters, in the following order:

*First.* Of the nature of the disease, and of its symptoms.

*Second.* Of its causes.

*Third.* Of the state and composition of the atmosphere, which proves the immediate cause of the hospital gangrene.

*Fourth.* Of the means of preventing that state of the atmosphere, or of correcting it the more speedily and efficaciously, if it is already formed.

Chapter I.—§ 3. *Of the Nature of the Disease and its Symptoms.*—Although the disease in question has been known ever since hospitals existed, and particularly in times of war, yet physicians and surgeons have but recently directed their attention to it;* a precise history, founded on correct observations and conclusive experiments, is still a desideratum.

§ 4. Even the name which it bears announces that in general the nature of it is not sufficiently known. It is called *gangræna nosocomialis, humid gangrene*, or *hospital putrescence*; in German, *hospitaal brand*; in Dutch, *hospitaal versterving*.

The ingenious Richerand† was the first to remark the vagueness of these denominations. In fact, although this disease threatens the gangrene and mortification of the part affected, when we do not succeed in arresting it, the evil may subsist for a long time without producing these dreadful effects. By an examination of its symptoms, it will appear that this gangrene is the effect of a general state of the system, which produces a peculiar local affection of wounds or ulcers; an affection, which, after having passed through different stages or periods, degenerates into mortification or gangrene, assuming all the symptoms of slow fever, and terminating with the life of the patient.

§ 5. In order to give an idea of the principal phenomena of this disease, I shall succinctly note the accidents which occur too often in the military hospitals among young men in high health, a few days after receiving even a slight wound, when they are conveyed into a room infected with the hospital gangrene.

A slight wound, which tears the skin, tends naturally to a prompt

---

* We believe our countryman, Dr. Rollo, of Woolwich, was the first who distinguished this ulcer as a morbid poison.—Edit.

† Nosographie Chirurgie, tom. i. p. 45, first edition; second edition, tom. i. p. 198.

 and and healthy suppuration; it does not affect the health of a young man, unless he is weakened by previous disease, excessive fatigue, or an irregular life. All the functions of the body are regularly performed, the ulceration diminishes daily, and, if left to itself, the cure would be certain; but, if the wounded man is conveyed into a room infected with hospital gangrene, the condition of the wound soon becomes worse.

Before this change in the wound is perceptible, the patient complains, sometimes a few hours after entering the hospital, of a most disagreeable sensation throughout the whole body, but particularly the arms and legs; lowness of spirits and disinclination for food follow; the tongue is dry and frequently covered with a yellowish coat; the thirst is inconsiderable when the appetite ceases; the pulse becomes unequal, small, and accelerated. This continued fever increases insensibly with irregular remissions, particularly towards evening. The countenance becomes wan, but the redness of the cheeks almost always denotes the access of the fever; the eyes become dull; the suppuration of the wound is altered; the secretion of pus sometimes diminishes; the edges swell, are painful to the touch, and, when seen through a magnifying glass, present eminences of a deep red, with depressed and often unequal edges. A species of inflammation now takes place on the wound; the pus is partly tenacious, like the white of an egg, and partly very fluid, transparent, and acrimonious: at the slightest touch the edges bleed. During this progress, there is often formed, at some distance from the ulcer, a circle of a deep red,* which extends more and more in concentric lines, the inflammatory state ceases, the eminences fall down, the colour of the edges becomes dull, the superficies is gangrenous, while a clear ichorous matter of a smell *sui generis* issues in greater or less quantity. The mortified surface is separated unequally and by portions; in different places irregular granulations arise, which run into gangrene; the evil extends by rapid strides, the circles most adjacent ulcerate, and those at a distance gradually run into them; the diseased surface is increased in depth as well as breadth. The gangrene attacks, in the first instance, the cellular texture, afterwards it seizes the muscles; even the tendons sometimes become ragged, as if they had been exposed for whole months to a slow putrifaction; the nerves and blood-vessels support themselves the longest. The bones also suffer greatly; they are denuded and gangrened by portions and in laminæ. I have in my collection the osseous part of the hand of a patient, who died of this disease: the superficies of all the metacarpal bones, and of the longest fingers, appear as if gnawed and separated more than a line in depth.

While the ulceration extends, emitting an insupportable smell peculiar to itself, while it appears under various forms, but chiefly

* This, we conceive, is the *specific distance* of a morbid poison, marked only by Mr. Hunter and the author of this paper, excepting in the cow-pox, where it is called the *areola.*—Edit.

in in angles, the general disease of the body is daily making new progress, the fever redoubles in intensity, the bodily strength is more and more exhausted, sometimes, but rarely, cutaneous eruptions arise in the form of small red spots. At length, the unhappy patient dies with all the symptoms of hectic fever.

§ 6. Such is the progress of this disease, when, without any sensible improvement and getting worse daily, it acquires that degree of force which destroys the patient. It is a peculiarity also, that when it proved mortal, its progress is suddenly arrested. But this appearance is deceitful, for the malady soon returns, and frequently even with redoubled force. Four, five, six times, or even oftener, the patient may be on the eve of convalescence, when repeated relapses at length bring on his dissolution.

§ 7. When the disease is disposed to heal, the crust which covers the pasty ulcer is then detached, the circles with which it is surrounded disappear, the surface is of a clearer red, the pus improves, the fever diminishes, the strength is restored, the appetite returns; the patient soon regains the condition in which he was when he was assailed by this dreadful disease, and a speedy cure is the consequence.

§ 8. The phenomena indicated differ in different subjects, and are modified by the constitution of the individual by previous or existing diseases, and by many other circumstances. If the strength of the patient is exhausted at the time of the appearance of this hospital disease, or if the food is of bad quality, the first period, viz. that inflammation (§ 5,) which is very distinctly observed in other cases is scarcely perceptible, and the last period which terminates in gangrene advances with great slowness. The gangrenous surface is by no means detached, or is separated but slowly and in thin portions.

Patients who have formerly used mercurial preparations, are more subject than others to a rapid and depascent gangrene.

§ 9. The disease differs particularly by an intensity commensurate with the degree of the contagion. This intensity shews itself by the rapid progress of the gangrene, and the disease in its first stage is often discovered by a difficulty attending the cure even of the most simple ulcers. Tenon has remarked, that, in general, wounds and ulcers are cured with much more difficulty in hospitals than in other places. Monstredon and Hunezoski are of the same opinion, and add, that this is the case more particularly in infirmaries communicating with those which receive patients under internal diseases.

Dessault expresses himself in the same way; and every attentive surgeon who has served in hospitals laments the same. The first stage of this hospital disease is manifested by the symptoms which constitute and accompany this difficulty of cure. The observation of those first and slight traces of the evil requires the most scrupulous attention on the part of the surgeon, who too often, without suspecting it, allows the favourable moment to slip when he might arrest the progress of the disease, and save his patients.

The

The inference is, that, from the instant we observe any difficulty in the cure of wounds and ulcers, accompanied by certain febrile symptoms, the cause of which we do not well know, it must be presumed that the patient is attacked with hospital gangrene, and, consequently, the most efficacious measures must be instantly taken for preventing the mischief.

§ 10. In order to mark more particularly the nature of the disease in question, let me be permitted to add some further reflection founded on experience.

1. The first indications of the disease are not observed in the wounds or ulcers, but in the whole body (§ 5.) It is only when the fever has shewn itself, that we perceive a change in the wound or ulcer; the disease, therefore, is not merely topical, it is a general disease, which nevertheless produces local affections. To the great prejudice of many patients, several surgeons think that this disease does not exist before they observe signs of gangrene in the ulcers themselves. This fatal opinion has cost many valuable lives, which might have been saved.

2. It is a febrile disease accompanied by symptoms quite peculiar to it. It has frequently a very great resemblance to the fevers commonly called putrid, *febres putridæ, typhi;* nevertheless, after minute attention, the difference will be perceptible. There is a peculiar conformity between it and the nosocomial fever, or typhus of prisons, described by Pringle, a disease of a complicated nature, and named by Pinal *adynamic & ataxic fever.* We shall soon see that both may originate from analogous causes, and that a contagion not being sufficiently strong for developing the true nosocomial fever among healthy persons, may, nevertheless, produce hospital gangrene. It is remarkable besides that the exhalations of putrid fevers, *febres adynamicæ, typhi*; although they produce hospital gangrene, the exhalations of the latter, while spreading the same contagion very powerfully among the wounded in the wards, nevertheless very rarely give rise to putrid fevers or typhi, among those who do not labour under wounds. The form of the ulcers attacked with hospital gangrene differs from that of all the other ulcers, as well as from every other species of mortification or gangrene. Pouteau says, (*Œuvres Posthumes*, vol. iii.) that it has no resemblance, except with the ulcers occasioned by the bites of a venomous serpent.

3. We may remark another peculiarity of this disease: the gangrene which it produces shows itself suddenly, and increases with a formidable rapidity. In fact, it is not rare, in the space of twenty-four hours, to find all the wounded in the same ward suddenly attacked with this disease, of which not the slightest vestige had been seen before. In the same way, a similar gangrene often takes, in a short interval, the most rapid strides, and speedily causes death: sometimes, however, the disease becomes chronic, and lasts for several months, without any remarkable change.

4. As the surface of the ulcers in which the first traces of hospital gangrene are remarked, presents a peculiar form and physiognomy (§ 5), which appears to be the result of an organization *sui generis,* it follows that we must seek for a peculiar organization, more especially in the state and composition of the pus which these ulcers yield.

The following are its chief properties, as far as I have observed them:—

*a.* The atmosphere which surrounds the patient is infected by the smell which the pus exhales, a smell which is so peculiar to it, that an experienced surgeon cannot mistake the disease, on entering the ward of an hospital where there is only a single wound attacked with this gangrene. This smell is always the same, notwithstanding the difference of subjects and temperaments. All other kinds of gangrene likewise exhale a smell which is peculiar to them, but which is clearly distinguished from the one in question.

*b.* In these ulcers, the degenerated pus is of two kinds (§ 5). Sometimes it is thicker and more tenacious than usual; sometimes it is clear, serous, and, as is commonly said, acrid. The first species is particularly remarked at the commencement of the disease; and the second at a more advanced stage.

In both cases, free and pure soda is evolved, which is not found in laudable pus. The colours of the curcuma, of the wood of fernambouc, and syrup of violets, are changed as with soda. If we dry the pus well by heat in a silver spoon, the alkaline principles remain; and, when we add some drops of muriatic acid, we obtain crystals of muriate of soda, as a sulphate of soda is formed by the addition of the sulphuric acid. The quantity of soda is here not only greater than in the ordinary pus, but it even exceeds that of the serosity of the blood. Those two kinds of altered pus, therefore, contain soda, but not in the same state. Lime-water is scarcely precipitated by that of the first species, when we take recent ulcerous matter, and carefully preserve it from the action of the air. The same precautions being taken, the second or serous species precipitates lime in the form of calcareous carbonate. In the first case, therefore, the soda is pure; in the second it is united with carbonic acid.

*c.* In laudable pus there is little albumen; it abounds, on the contrary, in the thick pus just mentioned. When we observe something like flakes in it, and it is gluey, a part of the albumen is already more or less coagulated, and the rest is coagulated by the acids and heat. We do not find albumen in the serous pus; a new proof that this pus is not produced by the transudation of the serum, but that it owes its origin to an altered secretion.

*d.* It seems that with this pus there is still extricated sulphuretted hydrogen gas and carbonic acid gas. I shall presently shew (§ 43.) that the pus of these ulcers communicates to the air these two species of gas.

*e.* The pus which flows from the ulcers is not a putrid matter, so long as the edges and superficies are not detached; but, as soon as this separation commences, there is a great tendency to putridity in every thing that comes from these ulcers.

Every

Every thing which becomes putrid favours the putrifaction of other putrescent substances. In my experiments, I mixed the pus of these ulcers, taken at different stages of the disease, with blood, recent flesh, and water. I exposed, under circumstances equally favourable to putrefaction, the vessels which contained this mixture, besides other vessels, which contained these bodies without the addition of pus. Repeated trials demonstrated that blood, recent flesh, and water, are not putrified more speedily when mixed with the pus taken during the first period of this disease, than when this addition is not made; but similar experiments convinced me that putrifaction is favoured by the pus collected in the last stage of the disease, particularly when the edges and surface are detached, and come away with the pus, like disgusting and fetid boiled flesh.

Since the pus emits a very strong smell before it becomes putrid, it is clear that this peculiar or disagreeable smell cannot be the produce of the putrifaction going on in the ulcer.

This is also proved by the clear and ichorous matter flowing in great quantity from the ulcers after the patient's death, but not emitting the putrifactive smell, as during life. If this bad smell was occasioned by putrifaction, it would necessarily be increased by death.

CHAPTER II.—§ 11. *Of the Causes of this Malady in general.*—Every person who has a wound, or an ulcer, is in a condition to receive this disease. The most trifling scratch on the skin is sufficient to occasion the hospital gangrene, if the atmosphere around the wounded person is infected with this contagion. No temperament, no other disease, whatever it may be, no circumstance which I know of, can preserve him; nevertheless, the disposition is greater in some patients than in others.

§ 12. The chief circumstances which increase the susceptibility are the following:

The vital powers weakened by previous disease, by deficient and bad food, by the long-continued breathing of a humid and unwholesome atmosphere, deprived of its oxygen; and by several other circumstances. The affections which depress the spirits, produce a wonderful effect—chagrin, anxiety, disquietude, and particularly a strong desire of re-visiting one's native country (*nostalgia*). Of late years, this last passion rendered several diseases mortal, and thousands of young men fell victims to it, among whom a great number were affected with hospital gangrene, the effect of constant broodings, which undermined their vital strength.

Different kinds of wounds even cause a stronger or weaker susceptibility.

The most disposed of all patients are those who have undergone the operation of trepan. Wounds in the arms and legs are more susceptible of hospital gangrene than those of other parts of the body; contused wounds, and those which have been made by a sharp instrument, are less so than gun-shot or splinter wounds.

§ 13. Previous to enumerating the occasional causes, it will be necessary necessary to determine, in the first place, whether we must consider hospital gangrene as an *epidemic*, or as a contagious disease, properly so called. If the former, it owes its origin to a state of the atmosphere; if the latter, it is produced by a miasma *sui generis*, which is communicated from one patient attacked by gangrene, or nosocomial putrifaction, to another, who soon exhibits all the symptoms of the same disease.

§ 14. Although I do not think it will be easy to find a medical officer who has observed this disease in a large military hospital, and who doubts its contagious nature, I consider it my duty, nevertheless, to demonstrate, in the first place, that this contagion is caused by a miasma *sui generis*, more particularly as the celebrated Richerand says, in express terms, "*The hospital gangrene is never contagious.*" For this purpose, it will be necessary to enter into some details respecting the manner in which this disease is propagated in one or more wards of an hospital.

§ 15. It is, in some measure, repugnant to our generally-received ideas, to admit a general cause without miasma, when this disease exists in, perhaps, only one of two hospitals in the same town, and is even prevalent only in one of the wards of those hospitals. The following observations will be still more decisive.

§ 16. Several physicians and surgeons have seen, with me, that this gangrene is communicated from a patient in bed, in the midst of one ward, to his neighbour, from the latter to a third, and so on to the farthest off, so that there remains not the slightest doubt respecting the propagation of the disease from one patient to another. It is true, that the nearest patient sometimes escapes for a certain time, and that the disease proceeds to the second or third; but then the disease attacks a little later him whom it had at first spared, and the reason is, that all wounds are not equally susceptible of receiving the infection.

§ 17. I have been a witness, more than once, in the military hospitals of Leyden, Utrecht, Nimeguen, and others, of a single wounded patient, coming from another hospital, whose wound, being affected with gangrene, or bearing the germs of it, diffused, at first, that disposition, as if issuing from a common centre, among all the wounded of a whole ward; or sometimes all the wounds and ulcers would be in the best condition, when suddenly the whole building was infected, unless prevented by the most prompt measures. This frequently happened at Leyden, to which place, for some years, the patients were brought from the hospitals of Alkmaar and the Helder, places which were frequently infected with this gangrene.

§ 18. In the hospital of Woerden, we were forced to receive a wounded man, attacked with the first symptoms of this gangrene, in a ward filled with fever patients, where there were sixteen beds ranged beside each other. He was placed at one end of the hall. In the same room, at the distance of fifty feet, lay a patient who had a large ulcer in the thigh, giving out laudable pus, and in a healing condition, during the three weeks that he had been in the hospital.

 Thirty-

Thirty-six hours after the admission of the above-mentioned gangrenous patient, the precursory symptoms of hospital gangrene appeared: soon the disease declared itself, but happily both were cured.

§ 19. At Leyden, at the end of the summer of 1798, in the French Military Hospital, the nosocomial gangrene prevailed in one of the lower wards, while the other patients, attacked with slight wounds, and placed above this room, in an airy attic, were free from the evil. The surgeon on duty thought it expedient to have a hole cut in the floor, in order to procure a vent for the foul air, in its progress through the roof. Thirty hours afterwards, the three wounded men who lay in the garret, nearest the aperture, were attacked with gangrene, which soon spread throughout the whole room.

In the foregoing cases, the contagion was spread through the atmosphere, and the miasma, according to every probability, was applied immediately to the ulcer. The following observation affords grounds to suspect that this disease may also be produced by the inspiration of the deleterious matter.

§ 20. In the month of August, 1805, I found, in one of the wards of an hospital at Amsterdam, four patients, whose wounds presented unequivocal symptoms of gangrene. The infection did not reign in the other wards. The rest of the patients, leaving only those four, of the above ward, were removed to other rooms, with the necessary precautions. Nevertheless, the number of the wounded became so considerable, that on the following day it was necessary to place two in the above room. Those two patients had each an ulcer of a good kind: one was situated above the calf of the left leg, and the other in the inside of the thigh. They were dressed without side of the room, nearly in the open air; and the dressings were covered with a wet bladder, so that the air of the room where they were to be placed could not be applied immediately on their ulcers. The dressing was carefully renewed, in the same manner, withoutside of the room, twice every twenty-four hours. Nevertheless, the fever which precedes gangrene seized the first patient twenty or twenty-two hours after his admission into the room, and the second nearly thirty hours afterwards, and both were attacked with gangrene.

§ 21. All who have described this disease, say, that it is communicable by the pus of ulcers, which are affected with it, and particularly by whatever may be impregnated with this pus, such as lint, linen, mattresses, blankets, sheets, &c. All this has been, alas, but too often confirmed by my own experience! The usual washing and bleaching of linen is not sufficient to annihilate the power of the contagious matter. In the year 1799, a quantity of lint was purchased in France, to be distributed in the various hospitals of Holland. Wherever it was used in dressing ulcers, a very violent hospital gangrene was the consequence. The fact was inquired into, and it was found that in the place from which the lint had been brought, they were in the habit of washing and bleaching the bandages and other articles used in the dressings of large hospitals, in order to arrange and sell them for new lint. These bandages are generally impregnated with pus. What an infamous fraud! This shews that washing and bleaching are not sufficient to destroy the miasma.

The celebrated Pelletan saw hospital gangrene occasioned by the employment of lint which had been kept several years locked up in boxes in the Hotel Dieu.

It has been remarked by Pouteau and others, that hospital gangrene has been manifested frequently in patients by the application of surgical instruments which had touched an ulcer infected with this evil.

§ 22. I conclude, from all I have said, from paragraph 15 to 21 inclusive, that hospital gangrene, and the disease which directly induces it, are contagious, so that they may pass from one patient to another, by means of contagious matter or miasma, which is yet to be examined; and, having now, I trust, sufficiently demonstrated that the atmosphere is charged with, retains, and propagates it, I shall next inquire into the origin of the infection, and under what circumstances it is developed.

§ 23. In the first place, there is no doubt that the ulcers affected with hospital gangrene have the property of producing the miasma in great abundance, so as to infect the atmosphere with it immediately. Thus, ulcers of the hospital gangrene have this in common with the ulcers produced by any other contagion, such as syphilis, small-pox, &c. that they give a pus *sui generis*, in which there exists, and is multiplied, *ad infinitum*, a contagious specific matter. See § 10.

§ 24. In the second place, the hospital gangrene is produced by the exhalations of all kinds of ulcers which emit an infectious smell: Such are, in particular, the cancerous, the scorbutic,* and the syphilitic, of an extensive surface; in short, every gangrene, and even every ulcer, which gives out a great quantity of pus, although of a good kind, may originate it. This contagious matter is equally produced by the exhalations of internal maladies, which tend to putrescence, or favour it by their secretions: such are putrid fevers, *typhi, febres adynamicæ*, and, more or less, all the fevers which give rise to abundant exhalations.

It is true, that by these general diseases the hospital gangrene is engendered less quickly among the wounded than by the exhalations of ulcers already affected with this evil; nevertheless, the baneful influence which they exercise is easily recognised. We may remark almost every day, that a patient is cured with more difficulty when he is in the vicinity of a person affected with one or other of the diseases mentioned, whether external or internal.

* It should be remarked, that, on the continent, by scorbutic is usually meant all the diseases, whether local or constitutional, which are induced by those diseases that excite hospital or camp fever.—Edit.

This

This difficulty of cure is the first symptom of an approaching gangrene. All surgeons have observed that this is proved by the exhalations of all the above diseases; and, if it were necessary, I could cite a multitude of examples. In the year 1811, the hospital gangrene broke out in the principal ward of the military hospital at Utrecht, and spread rapidly. The origin of the disease was found in an ulceration produced by a blister, which became fetid in a patient who had been carelessly dressed by an assistant-surgeon. This agrees with what Baglivius says in his treatise, *De usu et abusu vesicantium.* In 1799, a patient, attacked with a dreadful cancer in the cheek, passed the night in the hospital at Leyden, confounded with a great number of wounded: immediately we saw all the precursors of hospital gangrene.

It is well known that dissecting instruments soiled with the putrid matter of dead bodies, make wounds which easily change into malignant ulcers. In 1794, an assistant was infected in this way, in the Hanoverian military hospital near Leyden; although the wound was inconsiderable, on the second day the symptoms were so alarming, that he was placed among the wounded. The fever, and all the phenomena described in § 5, were manifested; finally, hospital gangrene followed, and was speedily communicated to the two nearest patients.

§ 25. In the third place, hospital gangrene is engendered by every discharge capable of producing putrefaction, particularly if an infectious smell is diffused.

Nothing is so noxious as the putrefaction of animal matter, and particularly pus, although, when fresh, the latter is in no way dangerous. Thus, when the heat of summer favours putrefaction, hospital gangrene sometimes shews itself suddenly, and in a very eminent degree, from the surface of a large ulcer not being cleaned with sufficient care, or because the dressing has been too long delayed, as must often happen in military hospitals.

It is also worthy of remark, that the bad smell of excrements is less noxious than disagreeable. I have frequently remarked, particularly in France, that the wards of the wounded were filled with a most insupportable smell of this kind, without any augmentation of the disposition to hospital gangrene.

The following is another fact in support of my assertion. M. Wyckerheld Bisdom requested me, a few years since, to visit the House of Correction, over which he is superintendant, in order that I might suggest any improvements. I found, among other defects, that in some places the sewers exhaled a smell so strong, that no person could approach them without repugnance; and even the prisoners were constantly affected with ophthalmia, which destroyed their eye-brows and eye-lashes. This smell did not, however, seem to injure their health; and we saw ulcers on the legs, without any trace of hospital gangrene.

§ 26. Finally, hospital gangrene is soon declared in places where the air enters with difficulty, and is never renewed; in short, under the same circumstances which, rendered more energetic, would cause cause the gaol-fever. Thus, in 1799, I saw, in the Hospital of the Bicetre of Paris, wounds affected with hospital gangrene.

In the House of Correction at Arnheim, eight men were shut up in a small yard, where the air was very bad. Seven were in good health: the eighth, when first confined, had a common ulceration in the leg; a short time afterwards, when I visited him with the celebrated surgeon Van Wy, hospital gangrene was manifest.

§ 27. From all that I have said from section 23 to 26 inclusive, I conclude that the miasma *sui generis* of hospital gangrene is engendered in the ulcers affected with this disease, and escapes from them; that all the substances which have a tendency to putrefaction communicate to the atmosphere such exhalations as afterwards produce the same disease. It must be added, however, that the peculiar odour of hospital gangrene is not observed in any state of atmospherical corruption, at least, if this degeneration does not already exist in an ulcer.

§ 28. From all that precedes, it is easy to deduce the circumstances which favour hospital gangrene, although, by themselves, they are incapable of producing it. We must here place in the first rank every thing that favours the putrefaction of animal substances, such as heat and humidity. It is in low, obscure, humid, and badly-aired places, and where the rays of the sun do not penetrate, that the disease first originates, and where it is cured with most difficulty. Every foreign exhalation tending to putrefaction is also very noxious.

The disease is also favoured by the use of beds too low, or when they are placed on the floor itself. The bottom of the bed ought to be at least 14 or 16 inches from the floor. In the following chapter I shall point out the reasons.

Every thing that weakens the body concurs to produce the disease. An unwholesome or meagre diet occasions the most alarming consequences. This explains why hospital gangrene was much more frequent in the French hospitals than in ours, before the French hospital system was introduced among us.

The following observation will not be misplaced here. In 1799, there was a great number of wounded Russians and Dutch in the hospital at Leyden. At first, both had the same food: speedily, however, the wounds of the Russians appeared to be gangrenous, and the fractures would not unite. We discovered the reason in the food, which suited the Dutch, not being strong enough for the Russians. Their quantity was, therefore, considerably increased: no more broth was distributed, but meat, potatoes, black and coarse bread, onions, plenty of salt, geneva, &c. Their condition was thus speedily ameliorated, and they were cured like the rest.

Richerand adds, that storms favour the development of this disease: for my part, I never could verify this assertion.

*(To be continued in our next.)*

Our readers will perceive by our first article, with which Mr. Yeatman has honoured us, that there is much contrariety in the opinions concerning the winds known in different parts of the world by the terms, Levanters, Siroccos, Harmattans, or Lestes. Whether these all arise from the same source, and are only differently modified by the sea and land they pass over, is not yet ascertained; we, therefore, earnestly solicit the communication of our more distant correspondents in different in parts of the world, that, by a just comparison of all the accounts, the fairest conclusions may be drawn. Meanwhile we insert short extracts from Sir J. Fellowes and others, tending to shew that the subject still wants further elucidation. Our readers may recollect some remarkably hot and dry days in the spring of the present year, when the wind was easterly, usually a cold though dry point. Can any of them offer a conjecture, whether these partake at all of the Levanters, &c.? If gentlemen who correspond with their friends in Gibraltar, Madeira, or Cadiz, would be careful in registering the periods when these winds occur, they might at once ascertain how far the times correspond with our hot south-easterns.

*Extracts from Sir James Fellowes's Reports of the Pestilential Disorders at Andalusia, &c. &c.* p. 12 & seq.

"Whilst this Levanter continues, the neighbouring mountains are scarcely to be seen, owing to a heavy dark mist which covers their surface. This wind is so dry and hot that it scorches the tender plants, and occasionally parches up the ripening corn, thus depriving the industrious husbandman in a few days of the reward of as many months' toil and labour.

"The effect of this terrible wind is not less remarkable upon animal life than upon the vegetable creation. The whole circulating system is influenced by it in a very extraordinary manner, the fibres become irritated, the quality of the bile itself is altered, and the most pacific, quiet temper is rendered irritable; so that quarrels, wounds, and assassinations, are said to be never more frequent than during the prevalence of a Levanter, which, in the province of Andalusia, is called the Wind of Discord.*

"It

* "Townsend, in his Journey through Spain in 1786 and 7, p. 439, speaking of Cadiz, has the following remark:—'Few places are more healthy than Cadiz; yet, when the Solano, or south wind blows, which comes to them over the scorching plains of Africa, having only the intervention of a strait, all the passions are inflamed, and during its prevalence, the inhabitants who are most irritable, commit every species of excess.'

"This statement, like those of most travellers who have undertaken to delineate the Spanish character, is to be received with great caution. Although the irritation occasioned by this wind displays itself sometimes in acts of outrage among the lower orders,

I have

"It is a vulgar opinion at Cadiz, that the Levant wind is salutary; but the best practitioners have observed, that, whilst it continues, all the complaints produced by a morbid state of the bile very frequently occur, especially cholera morbus, bilious diarrhœas, &c.; and that in general it so affects the human body, and occasions such debility, uneasiness, and apathy, as to unfit men for their ordinary occupations.

"Violent pain and giddiness are often brought on by this wind, and the fatal apoplexies which have occurred in Cadiz, are said by Gonzales and other physicians, to have taken place generally during its prevalence, owing to the powerful excitement induced by it, and in those subjects, whose moderate way of living exempted them from all suspicion of excesses, such as might have been supposed to have brought on the attack.

"It has been shewn from experience, that the Levant wind has always had a considerable influence over the Andalusian fever; the disorder has usually made its appearance in Spain, after the prevalence of easterly winds; this fact, observes Gonzales, although not sufficiently conclusive to enable us to account for the production of the disease, is, however, strong enough to induce us to consider it as one of the most powerful of the exciting causes.

"This destructive fever certainly became aggravated, more violent and unmanageable, and appeared always to be more fatal during the prevalence of a Levanter.*

---

I have observed, generally, the disposition and temper of the inhabitants of Cadiz, and of every other part of Spain that I visited, to be mild, friendly, and good-humoured; and hence I am more inclined to approve of a subsequent observation of this writer, where he says, 'for the pleasures of social intercourse, I did not meet with any city more agreeable than this. As all nations are here assembled within narrow limits, by their mutual intercourse they soften each other's manners, and as, notwithstanding the late shock, commerce flourishes in a degree, with its never-failing attendants, wealth and hospitality, a stranger may pass away his time with the highest satisfaction to himself.'"

* "The Levant wind, which affects the inhabitants of Cadiz, blows from the southward of east; and it would appear, that, by passing over a larger tract of the opposite coast of Africa, and a considerable portion of the land in Spain, it becomes drier and hotter than at Gibraltar, where it is also hot, but moister; this may be owing to the course of the wind, which sweeps, as it were, the surface of the Mediterranean sea, and has little land to impede its progress to the rock.

"At Malaga, the Levant wind is rather refreshing than oppressive, for it blows due east, and seems to pass over a greater extent of sea, and is consequently moister; but there the north and north-west wind, called the Terral, or land-wind, is very hot and oppressive during the summer months."

"It

"It must be allowed, that the atmosphere of Cadiz is particularly moist; for the wind, from whatever quarter it blows, before it reaches the town, passes over and sets in motion a great expanse of sea, and becomes charged with the humidity thence arising."

In addition to the above, and in order to bring as much of the subject as possible in view, we take the liberty of adding the following short extract from our own Journal.

"My reason for being so particular in describing Mr. De Carvalhal's house [in Madeira], was, that you might be aware how directly it is exposed to every breeze from the south-east. Those hot winds called in Africa, Harmattans; in the Mediterranean, Levanters; and in Italy, the Siroccos; occasionally visit this island, which is, I believe, the westernmost point of land at which they ever arrive. I need hardly refer you to Lynd or Norris for an account of the Harmattan, to Brydone for the Sirocco, your own acquaintance among such as have visited the Mediterranean will refresh your memory sufficiently. It is enough for me to give you some account of what we call a *Leste* in this country.

"Whenever the wind blows from the south-east, we have, for the most part, warm and dry weather, but at certain periods there is a kind of gloomy serenity in the air. The sun seems less powerful, being obscured by a general haziness in the atmosphere, yet the heat is much greater; and such is the universal dryness, that our mahogany card tables are warped, delicate pieces of inlaying fall out, and sheets of veneering, which cover larger furniture, blister and break off. The mucus of the eye is so soon exhausted, that a painful kind of stiffness is felt, accompanied with a sensation like scratching, whenever the eye-lids are brought over in order to restore the necessary moisture. Most, perhaps I might say all, of the hygrometers in the island, constructed on the principle proposed by Dr. Franklin, have become useless, probably by never recovering from the injury they sustained during these winds. Those I received from Mr. Lane are too limited in their scale to show any thing like the dryness of these winds.

"You will readily comprehend, that, though the valley in which Funchall is situated, is warmer than most other parts of the island which enjoy the benefit of the sea-breeze, yet, as we derive this advantage from being sheltered from every wind but the south, so, when a *leste* prevails, it is somewhat tempered by the interruption of our eastern hills. Hence *Palheiro do Ferreiro* being the first high lands exposed to these currents, receives its full shock. At the time the subjects were inoculated, we had a more violent and longer-continued *leste* than I have ever experienced. The natives complained more of it than the English, and were apprehensive of very serious consequences to their vines; many of these suffered exceedingly, and even some of the oranges and hardier chesnut trees were considerably blighted.

"If the *leste* continues long, the thermometer hovers about 80°, rarely rarely exceeding it in the middle of the day, and seldom falling much lower early in the morning. This heat might be tolerable, if we derived any advantage from our breezes; but at these times the air itself conveys the fuel. In the town it seems a little tempered by our Inbat from its passage through the Gullies; but at the Palheiro do Ferreiro they have not even this relief; and at the time I am speaking of, the air absolutely seemed to scorch.

"In the midst of all these inconveniencies, some advantages are derived from the *lestes*. Invalids who resort hither, particularly for pulmonary complaints, find a relief which so animates them, that they usually express a wish for a perpetual *leste*. But this leads me to Norris's account of these winds in Africa, part of which I shall transcribe.

"'So far (says our author) its effects on the animal and vegetable world are very disagreeable; but it is also productive of some good. The state of the air is extremely conducive to health; it contributes surprisingly to the cure of old ulcers and cutaneous eruptions. Persons labouring under fluxes and intermittent fevers generally recover in a Harmattan; and they who have been weakened and relaxed by fevers, and sinking under evacuations for the cure of them, particularly bleeding (which is often injudiciously repeated), have their lives saved in spite of the doctors. It stops the progress of epidemic diseases;—the small-pox, fluxes, and remittent fevers, not only disappear, but they who are labouring under them are almost sure of a speedy recovery. Infection is not then easily communicated. In the year 1770, I had above 300 slaves on-board a ship in the Whydah Roads, when the small-pox appeared among them. The greater part of these were inoculated before an Harmattan came on, and about 70 underwent that operation after it sat in: the former got very well through the disorder; none of the latter had either sickness or eruption. We thought we had got clear of the disorder, but in a few weeks it began to appear among these seventy. About fifty of them were inoculated a second time; the others had it in *the natural way*. An Harmattan came on, and they all recovered, except one girl, who had a malignant ulcer on the inoculated spot, and afterwards died of lock-jaw.'—*Norris's Memoirs*, and *Lit. Mag.* vol. iii. 254."

We have already requested the communications of our correspondents on this important branch of medical meteorology. The reader will now perceive an additional motive connected with our inquiry into the doctrine of contagion. By the African account, it appears that the Harmattan and Leste arrest the progress of contagion, and even of contagious as well as epidemical diseases. In Cadiz, the Levanters are said to increase the force of these maladies, and to give rise to others. It is not our intention to hint at any inaccuracy in either history, well aware of the great uncertainty in whatever relates to temperature, aspect, and habit; but the whole proves how much is still to be learned, and that it can only be accomplished by numerous and well-authenticated records.

